# Special Issue “Neuromuscular Disorders in Children and Adolescents”

**DOI:** 10.3390/children9040558

**Published:** 2022-04-14

**Authors:** Rudolf Korinthenberg

**Affiliations:** Research Ethics Committee, University of Freiburg, 79106 Freiburg, Germany; rudolf.korinthenberg@uniklinik-freiburg.de

Our call for contributions in early 2021 resulted in 10 peer-reviewed publications by the end of the year covering a wide range of topics in the field of neuromuscular diseases in children and adolescents. The term “neuromuscular disorders” includes acquired and inherited diseases of the peripheral nervous system affecting structures such as the spinal motor neurons and sensory root ganglia, cranial and peripheral nerves with their axons and myelin sheaths, neuromuscular synapses and muscle fibres. The articles in this Special Issue deal with the aetiology, diagnostics and treatment of the resulting diseases.

Rudolf Korinthenberg and colleagues from Germany report on their practice guideline on the differential diagnosis of acquired and hereditary neuropathies in children and adolescents. This guideline is based on a structured consensus process of experts in the field delegated by the participating medical–scientific societies in German-speaking countries. The guideline contains detailed recommendations for the diagnostic work-up of such neuropathies, including the ever-growing field of genetic neuropathies of the Charcot-Marie-Tooth type [1].

Agnieszka Cebula and her colleagues from Poland provide an overview of the current state of knowledge and application of the ultrasound elastography of muscles. This relatively new imaging technique for the qualitative and quantitative assessment of tissue elasticity is already used to examine various organs in adults, but experience in children is still limited. Analysis of the existing literature has been hampered by widely varying protocols in terms of instrument settings and probe positions. However, once the methods have been standardised, the authors expect this non-invasive method to be of great benefit for the diagnosis and follow-up of both natural history and treatment effects in diseases as diverse as muscular dystrophies and cerebral palsy [2].

Christina T. Rüsch and colleagues from Switzerland report on a large cohort of 38 children with carpal tunnel syndrome. Unlike in adults, the aetiology in these children was rarely idiopathic, but mostly secondary to tumours; vascular malformations; and—in these specialised centres—metabolic diseases, such as mucopolysaccharidoses. Electrophysiological examinations and nerve ultrasound were successful in establishing the diagnosis [3].

Kyung-Sun Park from Seoul, Korea, investigated two approaches to genetic analysis of Pompe disease in Korean and Japanese patients and in genome databases of the respective general populations. Approximately 50% of the pathogenic or likely pathogenic variants found in unaffected carriers were also found in affected patients with Pompe disease. The carrier frequency of Pompe disease in Koreans and Japanese patients was estimated to be 1.7% and 0.7%, respectively, and the predicted genetic prevalence was 1:13,657 and 1:78,013, respectively [4].

Katherine D. Mathew and her colleagues from the MD STAR network, USA, report on the characteristics of participants in clinical trials with Duchenne muscular dystrophy (DMD). DMD is the most common and one of the most severe neuromuscular diseases in children and adolescents. New treatment approaches have been under development for several years. For the planning of such clinical trials, it is important to assess the feasibility of the data presented and improve access to participation for those affected [5].

Adela Della Marina and her group from Germany report on the clinical course, myopathology and treatment challenges in two patients with autoimmune-mediated necrotising myopathy. This very rare disease can easily be confused with hereditary muscular dystrophy due to its clinical and pathological features. However, the possibility of immunosuppressive treatment makes correct diagnosis extremely important [6].

Minsu Gu and Hyun-Ho Kong from Korea report an improvement in fine manual dexterity in a small series of five children with spinal muscular atrophy type 2 after 18 months of treatment with nusinersen. While improvement in gross motor function has been repeatedly demonstrated, manual dexterity is also of great importance for daily tasks and quality of life. Such functional tests should be included in routine care and also in further large-scale clinical trials [7].

Meaghann S. Weaver and her colleagues from the USA studied several quality-of-life outcomes in 35 patients with varying severity of paediatric spinal muscular atrophy and their families. They report different positive and negative changes depending on the stage of nusinersen treatment, i.e., at initiation and during long-term treatment. This is important information for all professionals caring for this patient group [8].

Matthew A. Halanski and his co-authors from the USA report on the long-term follow-up of 32 children with SMA2 after posterior spinal instrumentation for progressive scoliosis. Scoliosis is a common orthopaedic complication in the natural history of SMA type 2, and its only effective treatment is surgery. However, secondary deformities may occur in the long term due to subsequent spinal growth. The authors describe postoperative deformities in the sagittal plane and their preconditions and consequences [9].

Akshata Huddar and co-workers from India report on three patients from two families suffering from ECEL1-associated distal arthrogryposis type D. These observations expand the phenotypic spectrum of this already known disorder, including clinical and MRI data. In particular, perinatal complications due to congenital contractures may lead to a misdiagnosis of perinatal damage [10].

Neuromuscular diseases encompass an enormous spectrum of acquired and genetic diseases, from Guillain-Barré syndrome to CMT neuropathy, and from congenital malformations to progressive muscular dystrophies. The correct and timely diagnosis and appropriate treatment are of paramount importance to patients and their families. While much of the treatment still relies on symptomatic measures to improve quality of life, there are new, more effective treatments for some of these diseases [see more on https://treat-nmd.org/resources-support/care-overview/] (accessed on 1 March 2022).
